# Supplementary figures and images for: Probing the molecular determinants of Ty1 retrotransposon restriction specificity in yeast
Source: PLoS Genet. 2025 Oct 9;21(10):e1011898. doi: 10.1371/journal.pgen.1011898 (PMC12530519; doi:10.1371/journal.pgen.1011898)

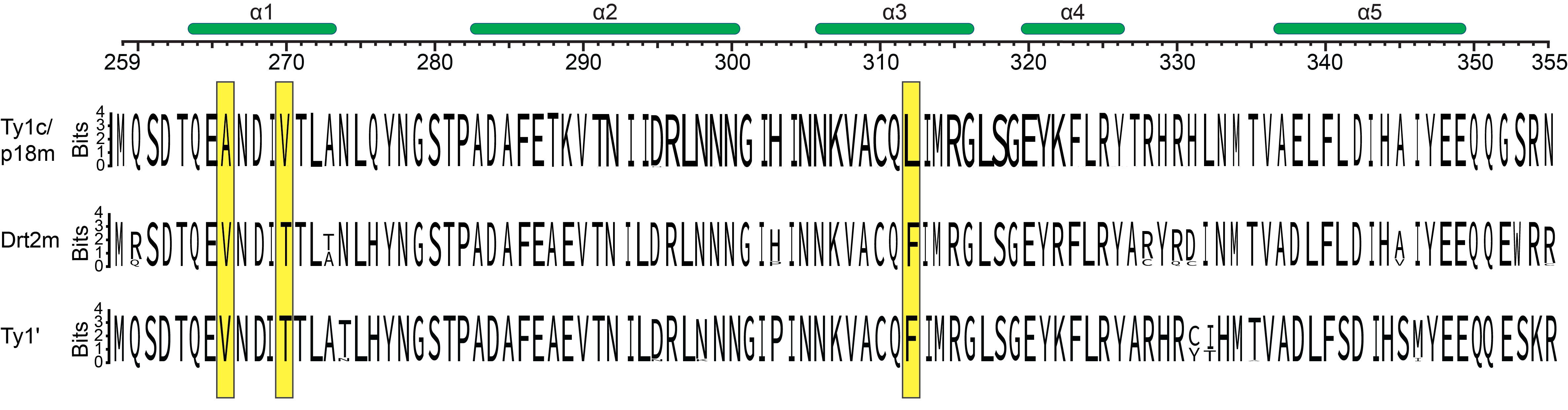

Supplement: S1 Fig — Amino acid coordinates and alpha helix positions (green bars) are indicated above the alignment. Three subfamily-specific residues are highlighted at positions 266, 270, and 312 (yellow boxes). Y-axis indicates sequence conservation measured in bits. (PNG) [file pgen.1011898.s001.png]

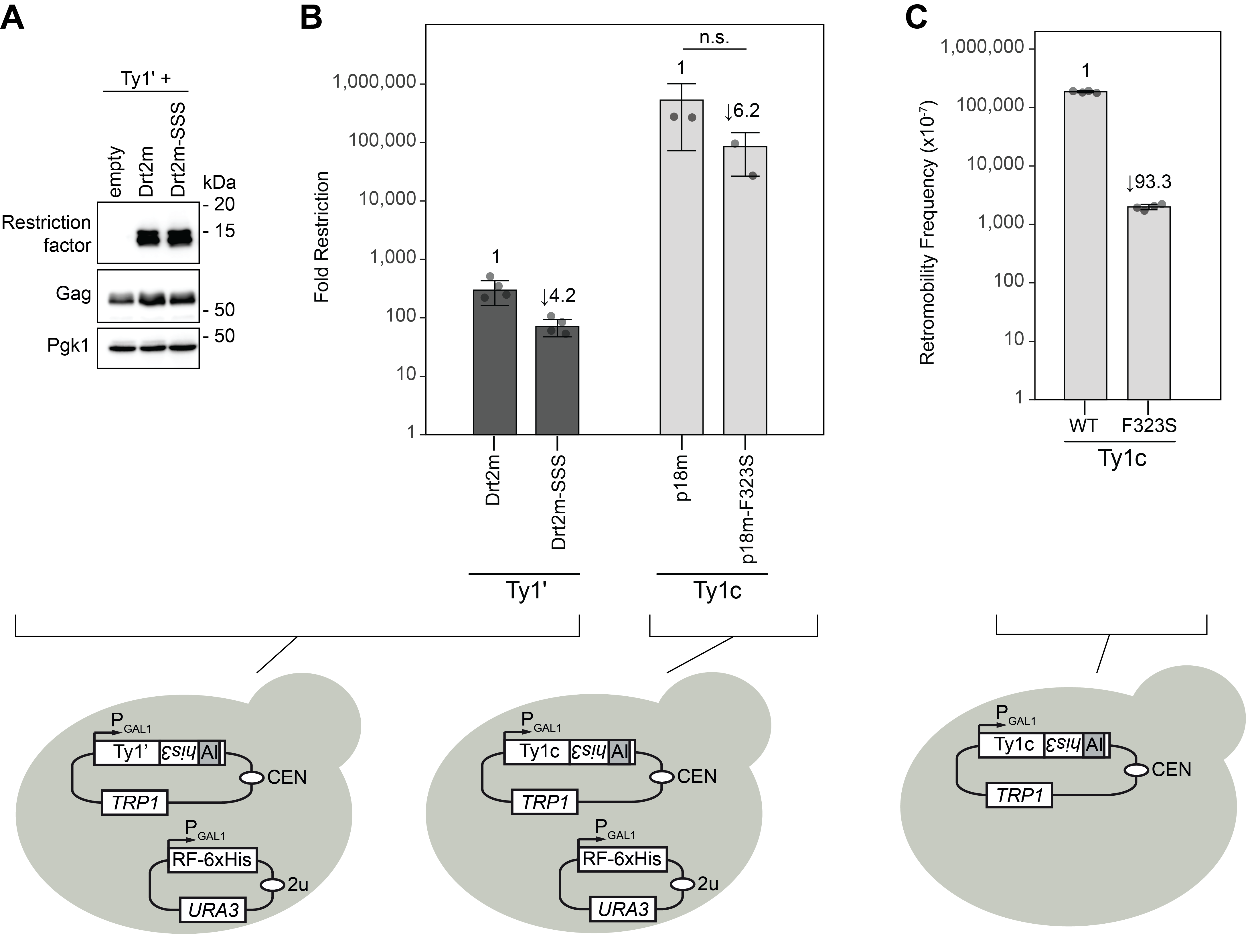

Supplement: S2 Fig — (A) Western blot of restriction factor and Gag expression. Protein extracts of galactose-induced cells were immunoblotted with an anti-hexa-histidine antibody to detect restriction factors and anti-p18’ to detect Ty1’ Gag. Pgk1 serves as a loading control. Migration of molecular weight standards is shown alongside the immunoblot. A representative image of at least 3 replicates is shown, original images of entire-gel immunoblots are provided in the Supporting Information. (B-C) (Upper) Quantitative mobility assay of galactose-induced cells. Each bar represents the mean of the four independent measurements displayed as points. The error bar center represents the mean of the four measurements and the error bar extent ± the standard deviation. Significance is calculated from a two-sided Student’s t-test compared with wildtype (n.s not significant, *** p < 0.001. Exact p-values are provided in S1 Table). In B, fold restriction is plotted compared to empty vector and fold-change in restriction compared to wildtype is indicated above the bars. In C, retromobility frequency in the absence of restriction factors is plotted. (Lower) Schematic illustrating the separate plasmids used to express the transposon and restriction factor (RF). The expression of each is driven by a galactose-inducible promoter from the GAL1 gene. The transposon is marked with the his3-AI retromobility indicator gene; histidine prototrophy requires retromobility. (PNG) [file pgen.1011898.s002.png]

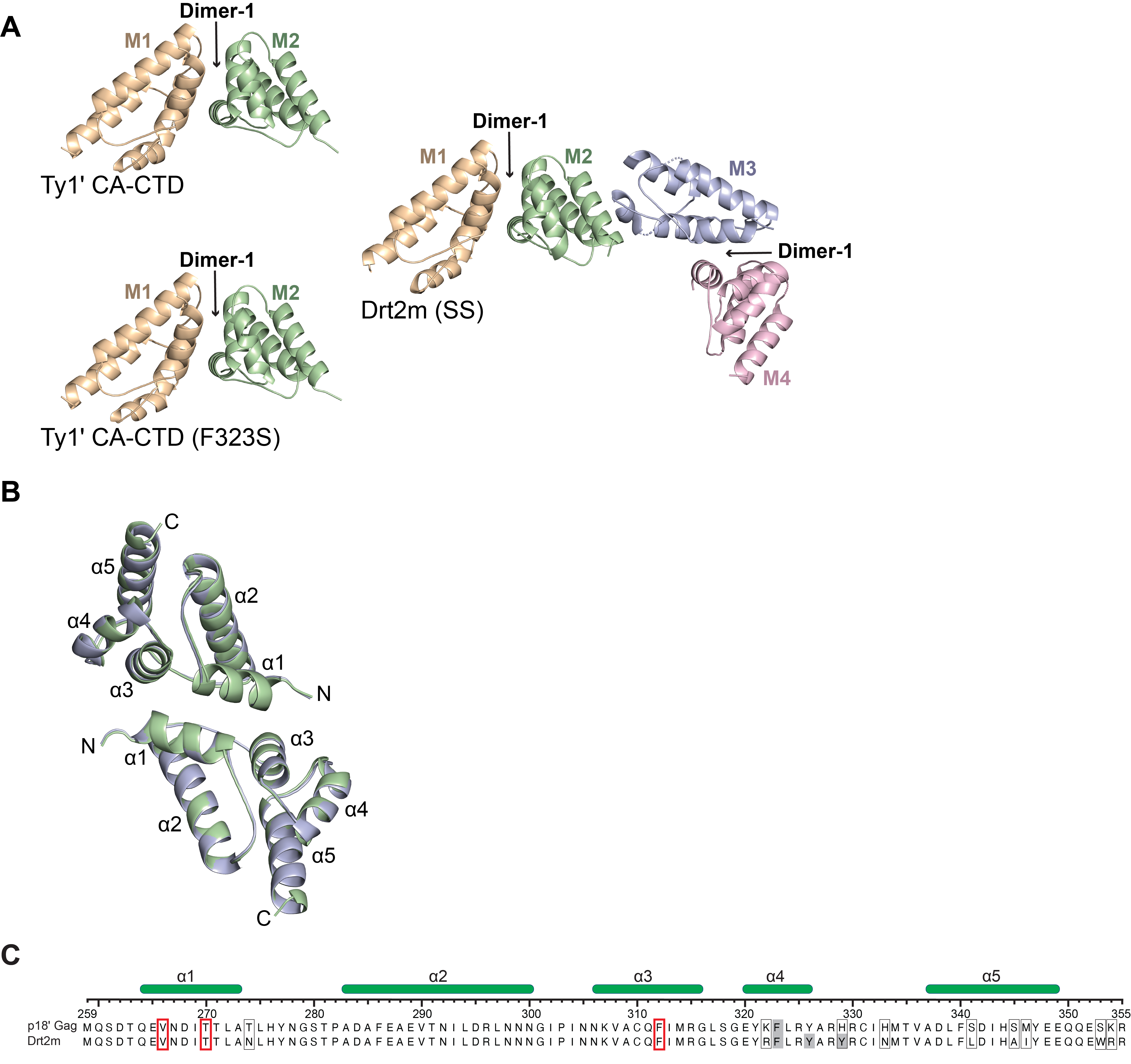

Supplement: S3 Fig — (A) The asymmetric units of the Ty1’ CA-CTD, Ty1’ CA-CTD (F323S) and Drt2m(SS) crystal structures. Protomers in each asymmetric unit are shown in cartoon representation colored wheat (M1), green (M2), pale blue (M3) and pink (M4). The equivalent Dimer-1 dimer interfaces are indicated with the arrows. (B) 3D structural superposition of Ty1’ CA-CTD (F323S) and Drt2m(SS) dimers. Backbone representations are colored green and pale blue respectively. Structures were aligned using 160 backbone Cα atoms, yielding an RMSD of 0.5 Å. Equivalent α-helices are labelled sequentially from N- to C-terminus. (C) Sequence alignment of Ty1’ Gag residues 259–355 and Drt2m. Numbering is according to the equivalent position in Ty1’ Gag. Positions of α-helices observed in crystal structures are indicated by the green bars above the alignment. Divergent residues are indicated by boxes, thick red boxes indicate Dimer-1 interface residues V, T, F where Ty1’ Gag and Drt2m are identical. Gray shaded residues indicate residues where Dimer-2 suppression serine mutations have been made. (PNG) [file pgen.1011898.s003.png]

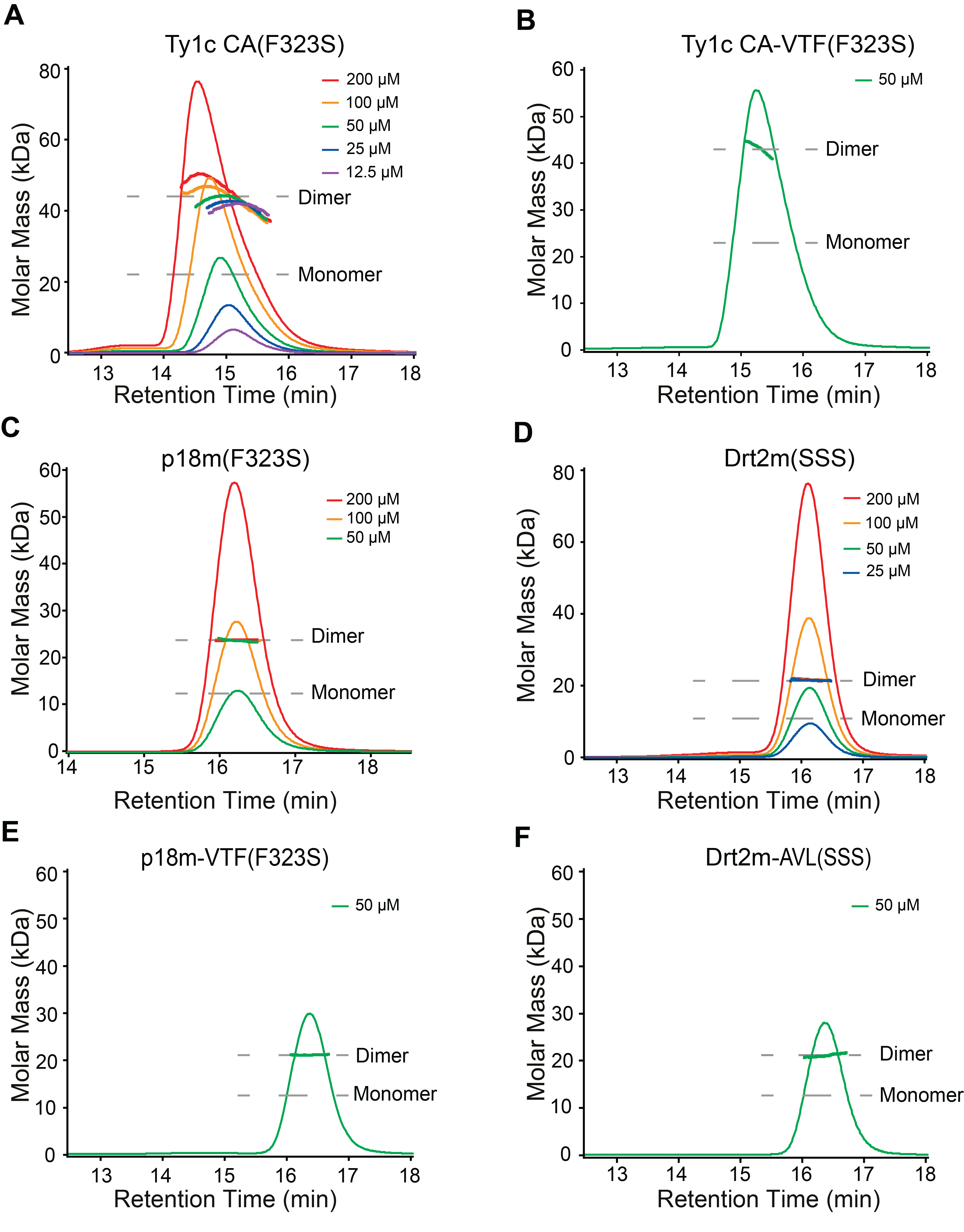

Supplement: S4 Fig — (A) Ty1c CA(F323S), (B) Ty1c CA-VTF(F323S), (C) p18m(F323S), (D) Drt2m(SSS), (E) p18m-VTF(F323S) and (F) Drt2m-AVL(SSS). In each panel dRI is plotted against retention time, the molar mass, determined at 1-second intervals throughout peak elution, is plotted as points and the sample loading concentrations are indicated; 200 µM (red), 100 µM (orange), 50 µM (green), 25 µM (blue) and 12.5 µM (violet). Monomer and dimer molar masses are indicated with the dashed lines. (PNG) [file pgen.1011898.s004.png]

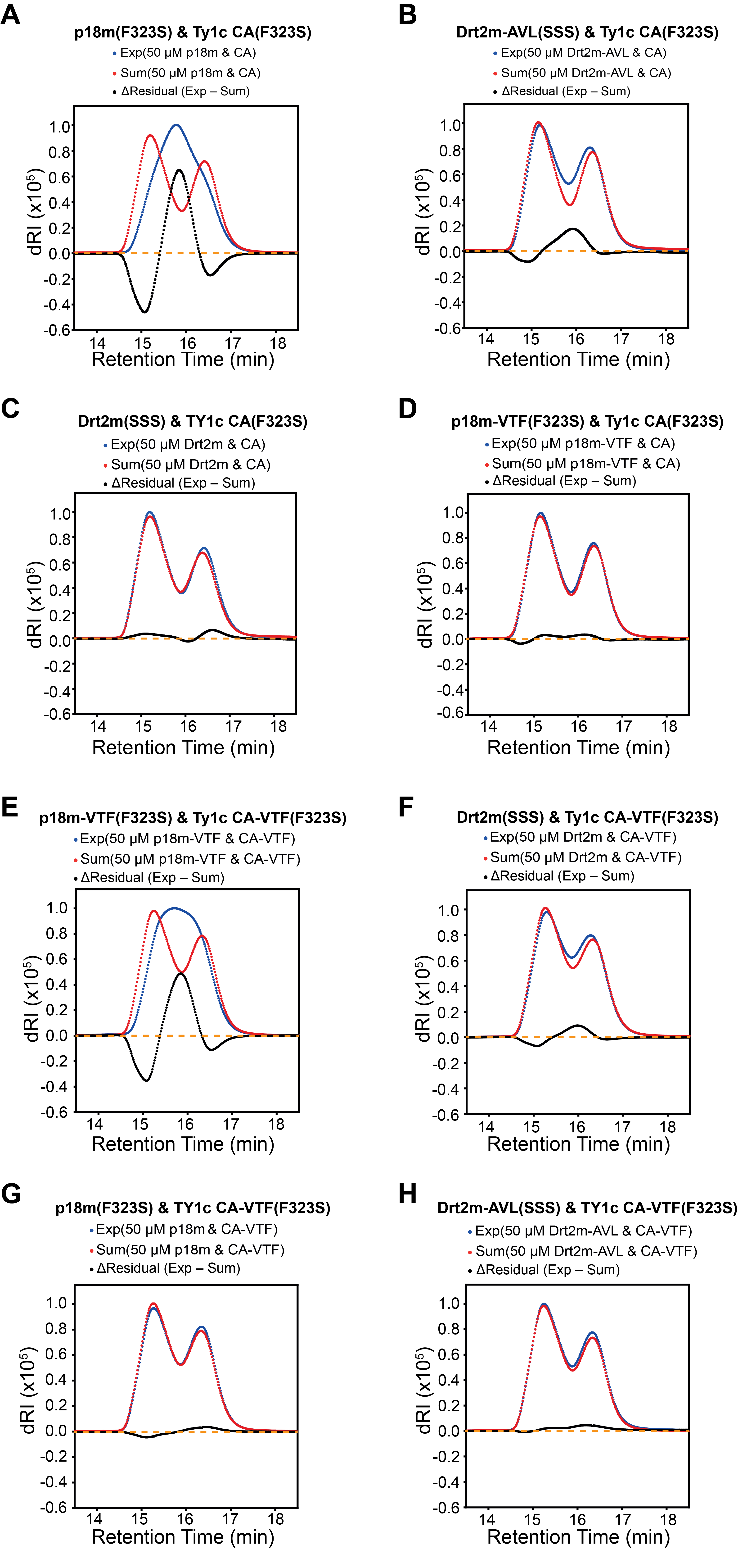

Supplement: S5 Fig — Dimer subunit exchange of (A-D) Ty1c CA(F323S) and (E-H) Ty1c CA-VTF(F323S) with p18m(F323S), p18m-VTF(F323S), Drt2m(SSS) and Drt2m-AVL(SSS) restriction factors are shown. In each panel, the experimentally measured dRI chromatogram (Exp) for 50 µM equimolar loadings of Ty1c CA(F323S) or Ty1c CA-VTF(F323S) with p18m(F323S), p18m-VTF(F323S), Drt2m(SSS) and Drt2m-AVL(SSS) restriction factors is shown in blue. The calculated sum chromatogram (Sum) derived by addition of individual 50 µM loading chromatograms for each pair is shown in red. The residual chromatogram (Residual) obtained after subtraction of the Sum chromatogram from the Exp chromatogram is shown in black. The orange dashed line indicates the baseline of zero residual. Integration over the peak elution envelope of the Exp, Sum and Residual chromatograms and application of equation-1 and equation-2 (see methods) was used to assess the degree of Ty1c CA(F323S) and Ty1c CA-VTF(F323S) subunit exchange with each p18m or Drt2m restriction factor. (PNG) [file pgen.1011898.s005.png]
