# Supplementary material for: Probing the molecular determinants of Ty1 retrotransposon restriction specificity in yeast
Source: PLoS Genet. 2025 Oct 9;21(10):e1011898. doi: 10.1371/journal.pgen.1011898 (PMC12530519; doi:10.1371/journal.pgen.1011898)
Supplement: S1 Table — (PDF) [file pgen.1011898.s006.pdf]

S1 Table. Retromobility frequencies

Data appears in figure:	Strain	Label	Retromobility Frequency (x10^{-7})	Std Dev (x10^{-7})	Fold Restriction	*p*-value[a]
Fig 1	DG4305	Ty1/*DRT2*	8,431.08	2,250.23	1.1	0.702
	DG4303	Ty1/*drt2Δ*	9,035.29	1,992.10	1	Reference
Fig 1	DG4259	Ty1'/*DRT2*	37.72	8.79	4.0	1.30E-04
	DG4304	Ty1'/*drt2Δ*	150.21	24.42	1	Reference
Fig 5	DG3739	Ty1/empty	257,649.67	58,228.94	1	Reference
	DG4147	Ty1/p18m	11.31	0.94	22,773	1.16E-04
	DG4574	Ty1/p18m-VTF	13,308.55	920.43	19	1.56E-04
	DG4573	Ty1/Drt2m	160,229.13	31,845.57	1.6	0.026
	DG4575	Ty1/Drt2m-AVL	57.46	8.65	4,484	1.16E-04
Fig 5	DG4581	Ty1-VTF/empty	276,712.33	22,592.36	1	Reference
	DG4582	Ty1-VTF/p18m	32,347.83	4,269.44	8.6	7.07E-07
	DG4584	Ty1-VTF/p18m-VTF	3.36	0.79	82,460	3.04E-07
	DG4583	Ty1-VTF/Drt2m	41.72	5.19	6,633	3.05E-07
	DG4585	Ty1-VTF/Drt2m-AVL	309,473.68	18,130.54	0.9	0.064
Fig 5	DG4576	Ty1'/empty	6,491.23	1,396.14	1	Reference
	DG4577	Ty1'/p18m	11,991.70	7,299.60	0.5	0.189
	DG4579	Ty1'/p18m-VTF	112.70	45.60	58	9.69E-05
	DG4578	Ty1'/Drt2m	50.58	6.79	128	9.15E-05
	DG4580	Ty1'/Drt2m-AVL	1,511.30	576.85	4.3	5.85E-04
Fig 5	DG4586	Ty1'-AVL/empty	16,231.16	1,824.80	1	Reference
	DG4587	Ty1'-AVL/p18m	239.44	14.94	68	2.21E-06
	DG4589	Ty1'-AVL/p18m-VTF	3,243.24	873.65	5.0	1.37E-05
	DG4588	Ty1'-AVL/Drt2m	516.22	97.25	31	2.47E-06
	DG4590	Ty1'-AVL/Drt2m-AVL	[b]	-	2,143	2.03E-06
Fig S2	DG4576	Ty1'/empty	5,707.62	1,018.87	1	3.08E-05
	DG4578	Ty1'/Drt2m	19.15	6.84	298	Reference
	DG4599	Ty1'/Drt2m-SSS	80.56	23.54	71	0.002
Fig S2	DG3739	Ty1/empty	199,099.10	28,125.81	1	7.76E-06
	DG4147	Ty1/p18m	0.38	0.43	528,807	Reference
	DG4350	Ty1/p18m-F323S	2.35	3.39	84,699	0.292
Fig S2	DG3735	Ty1 WT	185,656.73	7,522.35	1	Reference
	DG4348	Ty1 F323S	1,989.01	208.38	93	4.96E-09

[a]Calculated by two-sided Student's *t*-test; $p > 0.05$ are colored red.

[b]No retromobility events observed. Fold restriction and *p*-value reported for if one event had been observed.
