# Supplementary material for: Probing the molecular determinants of Ty1 retrotransposon restriction specificity in yeast
Source: PLoS Genet. 2025 Oct 9;21(10):e1011898. doi: 10.1371/journal.pgen.1011898 (PMC12530519; doi:10.1371/journal.pgen.1011898)
Supplement: S2 Table — (PDF) [file pgen.1011898.s007.pdf]

S2 Table. X-ray data collection and structure refinement statistics

	TY1' CA-CTD	TY1' CA-CTD (F323S)	Drt2m(SS)
**Data collection**	Diamond I03	Diamond I24	Diamond I04
Space group	P1	$P2_1$	C2
Cell dimensions			
a, b, c (Å)	31.59,34.33,45.33	36.23,52.75,45.96	91.76,38.83,111.21
α, β, γ (°)	101.64,96.35,109.28	90.00,108.54,90.00	90.00,96.65,90.00
Wavelength (Å)	0.97625	0.99990	0.97948
Resolution isotropic (Å)	43.59-1.60 (1.62-1.60)†	43.58-1.62 (1.79-1.62)	110.46-3.18 (3.54-3.18)
Resolution anisotropic principal axes (Å)	-	1.62/1.93/1.72	3.01/3.87/3.89
Unique reflections	18863 (361)	15110 (755)	4022 (202)
R_{meas} (%)	6.7 (152.4)	8.7 (107.3)	17.6 (149.0)
R_{pim} (%)	3.5 (83.3)	3.0 (46.5)	6.9 (57.6)
$CC_{1/2}$	0.999 (0.492)	0.997 (0.596)	0.997 (0.534)
$I/\sigma(I)$	20.0 (0.8)	10.8 (0.9)	7.2 (1.2)
Completeness (%)			
Spherical	82.6 (31.3)	71.4 (13.6)	59.2 (11.1)
Ellipsoidal	-	90.2 (45.1)	83.3 (50.9)
Multiplicity	3.4 (3.1)	7.8 (4.7)	6.4 (6.6)
**Refinement**	Refmac 5.8	Refmac 5.8	Phenix.refine
Resolution (Å)	43.58-1.60 (1.64-1.60)	43.57-1.62 (1.66-1.62)	45.57-3.18 (3.30-3.18)
Refl working/free	17855/978 (508/36)	14350/759 (49/2)	3821/191 (35/3)
R_{work}/R_{free}/Test set size (%)	18.1/20.2/5.2 (35.6/33.1/6.6)	22.2/26.8/5.0 (46.9/50.0/3.2)	29.9/33.9/4.8 (38.7/47.4/7.9)
No residues/atoms			
Protein	180/1473	181/1399	339/2313
Water	74	32	-
All	1547	1431	2313
B-factors ($Å^2$)			
Wilson	20.42	38.49	63.68
Protein	20.23	40.33	62.68
Water	36.1	39.2	-
Geometry			
RMSD Bond lengths (Å)	0.0076	0.0064	0.002
RMSD Bond angles (°)	1.375	1.261	0.446
Ramachandran Outliers (%)	0.00	0.00	0.00
Ramachandran Allowed (%)	0.57	1.69	1.24
Ramachandran Favoured (%)	99.43	98.31	98.76
Molprobity score (N number/percentile)	1.45 (7299/91st)	1.45 (711/89th)	1.24 (1547/100th)
PDB code	9RXW	9RXX	9RXY

†Values in parenthesis refer to the highest resolution shell.
