# Supplementary material for: Probing the molecular determinants of Ty1 retrotransposon restriction specificity in yeast
Source: PLoS Genet. 2025 Oct 9;21(10):e1011898. doi: 10.1371/journal.pgen.1011898 (PMC12530519; doi:10.1371/journal.pgen.1011898)
Supplement: S3 Table — (PDF) [file pgen.1011898.s008.pdf]

S3 Table Hydrodynamic parameters[a] and Sedimentation equilibrium data

Hydrodynamic Parameter	**p18(F323S)**	**Drt2(SSS)[b]**
[c]ν (mL.g^{-1})	0.725	0.722
[d]ρ (g.mL^{-1})	1.006	1.006
[e]η (x10^{2}) (g^{-1} cm^{-1} s^{-1})	1.031	1.031
[f]M_r	11,389	11,430
[g]ε_{280} (M^{-1} cm^{-1})	5,200	9,600
[h]dn/dc (ml g^{-1})	0.186	0.188
[i]j_{inc} (M^{-1}.cm^{-1})	31,320	31,432

p18m(F323S) Sedimentation equilibrium combined IF & Abs data 1-2-4 model				
C (µM)	22	45	93	22-93
[j]M_w kD	34.4	37.9	35.2	34.4-37.9
[k]$K_D^{(1-2)}$ (µM)	0.68	0.87	0.69	0.71
[l]$K_D^{(2-4)}$ (µM)	32.2	18.9	33.7	30.5
[m]rmsd	0.0019-0.0037	0.0027-0.0036	0.0028-0.0029	0.0021-0.0041
[n]n	6598	6455	5745	18798
[o]χ^2	0.308	0.426	0.329	0.407

Drt2m(SSS) sedimentation equilibrium combined IF & Abs data 1-2-4 model				
C (µM)	22	44	88	22-88
M_w kD	27.0	32.0	31.8	27-32.0
$K_D^{(1-2)}$ (µM)	0.46	0.50	0.48	0.48
$K_D^{(2-4)}$ (µM)	237	279	276	277
rmsd	0.0015-0.0018	0.0018-0.0024	0.0024-0.0025	0.0019-0.0024
n	3548	3466	3356	10370
χ^2	0.103	0.176	0.238	0.190

[a]Protein and buffer hydrodynamic constants referenced to 293 K; [b]triple Dimer-2 interface supressing mutant F323S/Y326S/Y329S; [c]Protein partial specific volume; [d]Buffer density; [e]Buffer viscosity; [f]Molar mass calculated from the protein sequence; [g]Molar absorbance extinction coefficient; [h]refractive index increment; [i]Molar fringe increment. [j]weight averaged molecular weight derived from multispeed analysis of individual samples using single species model; [k]monomer-dimer equilibrium dissociation constant determined from a global fit using three concentrations and three speeds to a monomer-dimer-tetramer self-association model; [l]Dimer-tetramer equilibrium dissociation constant derived from a monomer-dimer-tetramer self-association model; [m]root mean square deviation observed for each multi-speed sample when fitted individually and globally to a monomer-dimer-tetramer self-association model; [n]number of data points; [o]global reduced chi-squared from combined fitting of all multispeed data to a monomer-dimer-tetramer self-association model.
