# Supplementary material for: Probing the molecular determinants of Ty1 retrotransposon restriction specificity in yeast
Source: PLoS Genet. 2025 Oct 9;21(10):e1011898. doi: 10.1371/journal.pgen.1011898 (PMC12530519; doi:10.1371/journal.pgen.1011898)
Supplement: S4 Table — (PDF) [file pgen.1011898.s009.pdf]

S4 Table. Yeast strains used in this study

Used in Fig:	Strain	Genotype	Plasmids	Source
1	DG4305	DG4259	pBDG633	This study
1	DG4303	DG4259 *drt2::NatMX*	pBDG633	[1]
1	DG4259	[a]	pBDG1785	[1]
1	DG4304	DG4259 *drt2::NatMX*	pBDG1785	[1]
	DG3582	[b]		[2]
5, 6, S2	DG3739	DG3582	pBDG1534, pBDG1293	[2]
5, 6, S2	DG4147	DG3582	pBDG1534, pBDG1646	[3]
5, 6	DG4574	DG3582	pBDG1534, pBDG1819	This study
5, 6	DG4573	DG3582	pBDG1534, pBDG1758	This study
5, 6	DG4575	DG3582	pBDG1534, pBDG1818	This study
5	DG4581	DG3582	pBDG1820, pBDG1293	This study
5	DG4582	DG3582	pBDG1820, pBDG1646	This study
5	DG4584	DG3582	pBDG1820, pBDG1819	This study
5	DG4583	DG3582	pBDG1820, pBDG1758	This study
5	DG4585	DG3582	pBDG1820, pBDG1818	This study
5, S2	DG4576	DG3582	pBDG1697, pBDG1293	This study
5	DG4577	DG3582	pBDG1697, pBDG1646	This study
5	DG4579	DG3582	pBDG1697, pBDG1819	This study
5, S2	DG4578	DG3582	pBDG1697, pBDG1758	This study
5	DG4580	DG3582	pBDG1697, pBDG1818	This study
5	DG4586	DG3582	pBDG1821, pBDG1293	This study
5	DG4587	DG3582	pBDG1821, pBDG1646	This study
5	DG4589	DG3582	pBDG1821, pBDG1819	This study
5	DG4588	DG3582	pBDG1821, pBDG1758	This study
5	DG4590	DG3582	pBDG1821, pBDG1818	This study
S2	DG4599	DG3582	pBDG1697, pBDG1828	This study
6	DG4292	DG3582	pBDG1646	[3]
6	DG4570	DG3582	pBDG1758	This study
S2	DG4350	DG3582	pBDG1534, pBDG1747	[3]
S2	DG3735	DG3582	pBDG1534	[3]
S2	DG4348	DG3582	pBDG1749	[3]

[a] *Saccharomyces cerevisiae MATα ho::HygroMX ura3::KanMX flo8::G418A his3-Δ200hisG trp1-hisG* Ty-less

[b] *Saccharomyces paradoxus MATα gal3 his3-Δ200hisG trp1-1* ura3* Ty-less

References

1. Hannon-Hatfield JA, Chen J, Bergman CM, Garfinkel DJ. Evolution of a restriction factor by domestication of a yeast retrotransposon. Arkhipova I, editor. Molecular biology and evolution. 2024;41. doi:10.1093/molbev/msae050
2. Saha A, Mitchell JA, Nishida Y, Hildreth JE, Ariberre JA, Gilbert WV, et al. A trans-dominant form of Gag restricts Ty1 retrotransposition and mediates copy number control. Journal of virology. 2015;89: 3922–38. doi:10.1128/JVI.03060-14
3. Cottee MA, Beckwith SL, Letham SC, Kim SJ, Young GR, Stoye JP, et al. Structure of a Ty1 restriction factor reveals the molecular basis of transposition copy number control. Nature Communications. 2021;12: 5590. doi:10.1038/s41467-021-25849-0
