# Supplementary material for: Probing the molecular determinants of Ty1 retrotransposon restriction specificity in yeast
Source: PLoS Genet. 2025 Oct 9;21(10):e1011898. doi: 10.1371/journal.pgen.1011898 (PMC12530519; doi:10.1371/journal.pgen.1011898)
Supplement: S5 Table — (PDF) [file pgen.1011898.s010.pdf]

S5 Table. Yeast plasmids used in this study

Plasmid	Description	Markers	Source
pBDG633	pTy1*his3-AI*	*URA3/CEN*	[1]
pBDG1785	pTy1'*his3-AI*	*TRP1/CEN*	[2]
pBDG1534	pGTy1*his3-AI*	*TRP1/CEN*	[3]
pBDG1697	pGTy1'*his3-AI*	*TRP1/CEN*	[2]
pBDG1820	pBDG1534-Gag-A266V/V270T/L312F	*TRP1/CEN*	This study
pBDG1821	pBDG1697-Gag-V266A/T270V/F312L	*TRP1/CEN*	This study
pBDG1293	p*GAL*-Yes2	*URA3/2µ*	Invitrogen
pBDG1646	pBDG1293-p18m-6xHis	*URA3/2µ*	[4]
pBDG1819	pBDG1646-p18m-A266V/V270T/L312F	*URA3/2µ*	This study
pBDG1758	pBDG1293-Drt2m-6xHis	*URA3/2µ*	[2]
pBDG1818	pBDG1758-Drt2m-V266A/T270V/F312L	*URA3/2µ*	This study
pBDG1828	pBDG1758-Drt2m-F323S/Y326S/Y329S	*URA3/2µ*	This study
pBDG1747	pBDG1646-p18m-F323S	*URA3/2µ*	[4]
pBDG1749	pBDG1534-Gag-F323S	*TRP1/CEN*	[4]

References

1. Lee BS, Lichtenstein CP, Faiola B, Rinckel LA, Wysock W, Curcio MJ, et al. Posttranslational inhibition of Ty1 retrotransposition by nucleotide excision repair/transcription factor TFIIH subunits Ssl2p and Rad3p. Genetics. 1998;148: 1743–61. doi:10.1093/genetics/148.4.1743 (new 1)
2. Hannon-Hatfield JA, Chen J, Bergman CM, Garfinkel DJ. Evolution of a restriction factor by domestication of a yeast retrotransposon. Arkhipova I, editor. Molecular biology and evolution. 2024;41. doi:10.1093/molbev/msae050 (new 2)
3. Saha A, Mitchell JA, Nishida Y, Hildreth JE, Ariberre JA, Gilbert WV, et al. A trans-dominant form of Gag restricts Ty1 retrotransposition and mediates

copy number control. Journal of virology. 2015;89: 3922–38. doi:10.1128/JVI.03060-14 (new 3)

4. Cottee MA, Beckwith SL, Letham SC, Kim SJ, Young GR, Stoye JP, et al. Structure of a Ty1 restriction factor reveals the molecular basis of transposition copy number control. Nature Communications. 2021;12: 5590. doi:10.1038/s41467-021-25849-0 (new 4)
