# Supplementary material for: Probing the molecular determinants of Ty1 retrotransposon restriction specificity in yeast
Source: PLoS Genet. 2025 Oct 9;21(10):e1011898. doi: 10.1371/journal.pgen.1011898 (PMC12530519; doi:10.1371/journal.pgen.1011898)
Supplement: S6 Table — (PDF) [file pgen.1011898.s011.pdf]

S6 Table. Codon-optimised gene sequences

Construct	Sequence†
Ty1' CA-CTD (M259-Q351)	ATGCAGAGCGATACCCAAGAAGTGAACGATATTACCACACTGGCAACCCTGCATTATAACGGTAG<br>CACACCGGCAGATGCATTTGAAGCAGAAGTTACCAATATTCTGGATCGCCTGAATAATAACGGCA<br>TTCCGATTAATAATAAAGTGGCCTGCCAGTTTATTATGCGTGGTCTGAGCGGTGAATACAAATTTC<br>TGCGTTATGCACGTCATCGTTGCATTCACATGACCGTTGCAGACCTGTTTAGCGATATTCACAGCA<br>TGTATGAAGAACAGCCG
Ty1' CA-CTD (M259-Q351)(F323S)	ATGCAGAGCGATACCCAAGAAGTGAACGATATTACCACACTGGCAACCCTGCATTATAACGGTAG<br>CACACCGGCAGATGCATTTGAAGCAGAAGTTACCAATATTCTGGATCGCCTGAATAATAACGGCA<br>TTCCGATTAATAATAAAGTGGCCTGCCAGTTTATTATGCGTGGTCTGAGCGGTGAATACAAATCTC<br>TGCGTTATGCACGTCATCGTTGCATTCACATGACCGTTGCAGACCTGTTTAGCGATATTCACAGCA<br>TGTATGAAGAACAGCCG
Drt2m	ATGCAGAGCGATACCCAAGAAGTGAACGATATTACCACACTGGCAAACCTGCATTATAACGGTAG<br>CACACCGGCAGATGCATTTGAAGCAGAAGTTACCAATATTCTGGATCGCCTGAATAATAACGGCA<br>TTCCGATTAATAATAAAGTGGCCTGCCAGTTTATTATGCGTGGTCTGAGCGGTGAATATCGTTTTC<br>TGCGTTATGCACGTTATCGCTGCATTAATATGACCGTTGCAGACCTGTTTCTGGATATTCATGCCA<br>TTTATGAAGAACAGCAAGAATGGCGTCGTCCG
Drt2m(SS)	ATGCAGAGCGATACCCAAGAAGTGAACGATATTACCACACTGGCAAACCTGCATTATAACGGTAG<br>CACACCGGCAGATGCATTTGAAGCAGAAGTTACCAATATTCTGGATCGCCTGAATAATAACGGCA<br>TTCCGATTAATAATAAAGTGGCCTGCCAGTTTATTATGCGTGGTCTGAGCGGTGAATATCGTTCTC<br>TGCGTTATGCACGTTCTCGCTGCATTAATATGACCGTTGCAGACCTGTTTCTGGATATTCATGCCA<br>TTTATGAAGAACAGCAAGAATGGCGTCGTCCG
Drt2m(SSS)	ATGCAGAGCGATACCCAAGAAGTGAACGATATTACCACACTGGCAAACCTGCATTATAACGGTAG<br>CACACCGGCAGATGCATTTGAAGCAGAAGTTACCAATATTCTGGATCGCCTGAATAATAACGGCA<br>TTCCGATTAATAATAAAGTGGCCTGCCAGTTTATTATGCGTGGTCTGAGCGGTGAATATCGTTCTC<br>TGCGTTCTGCACGTTCTCGCTGCATTAATATGACCGTTGCAGACCTGTTTCTGGATATTCATGCCA<br>TTTATGAAGAACAGCAAGAATGGCGTCGTCCG
Drt2m-AVL(SSS)	ATGCAGAGCGATACCCAAGAAGCAAACGATATTGTTACACTGGCAAACCTGCATTATAACGGTAG<br>CACACCGGCAGATGCATTTGAAGCAGAAGTTACCAATATTCTGGATCGCCTGAATAATAACGGCA<br>TTCCGATTAATAATAAAGTGGCCTGCCAGCTGATTATGCGTGGTCTGAGCGGTGAATATCGTTCTC<br>TGCGTTCTGCACGTTCTCGCTGCATTAATATGACCGTTGCAGACCTGTTTCTGGATATTCATGCCA<br>TTTATGAAGAACAGCAAGAATGGCGTCGTCCG
p18m	ATGCAGAGCGATACCCAAGAAGCAAACGATATTGTTACCCTGGCAAATCTGCAGTATAACGGTAG<br>CACACCGGCAGATGCATTTGAAACCAAAGTTACCAACATTATCGATCGCCTGAATAACAACGGCA<br>TCCACATTAATAACAAAGTTGCCTGTCAGCTGATTATGCGTGGTCTGAGCGGTGAATACAAATTTC<br>TGCGTTATACCCGTCATCGTCATCTGAATATGACCGTTGCAGAACTGTTTCTGGATATTCATGCCA<br>TCTATGAAGAACAGCAGGGTAGCCGTAATCCG
P18m(F323S)	ATGCAGAGCGATACCCAAGAAGCAAACGATATTGTTACCCTGGCAAATCTGCAGTATAACGGTAG<br>CACACCGGCAGATGCATTTGAAACCAAAGTTACCAACATTATCGATCGCCTGAATAACAACGGCA<br>TCCACATTAATAACAAAGTTGCCTGTCAGCTGATTATGCGTGGTCTGAGCGGTGAATACAAATCTC<br>TGCGTTATACCCGTCATCGTCATCTGAATATGACCGTTGCAGAACTGTTTCTGGATATTCATGCCA<br>TCTATGAAGAACAGCAGGGTAGCCGTAATCCG
P18m-VTF(F323S)	ATGCAGAGCGATACCCAAGAAGTTAACGATATTACCACCCTGGCAAATCTGCAGTATAACGGTAG<br>CACACCGGCAGATGCATTTGAAACCAAAGTTACCAACATTATCGATCGCCTGAATAACAACGGCA<br>TCCACATTAATAACAAAGTTGCCTGTCAGTTTATTATGCGTGGTCTGAGCGGTGAATACAAATCTC<br>TGCGTTATACCCGTCATCGTCATCTGAATATGACCGTTGCAGAACTGTTTCTGGATATTCATGCCA<br>TCTATGAAGAACAGCAGGGTAGCCGTAATCCG
Ty1c CA (M-V169-N355)	ATGGTTCGTCCGCCTCCGATGCTGACCAGTCCGAATGATTTTCCGAATTGGGTTAAAACCTATATC<br>AAATTCCTGCAGAATAGCAACCTTGGTGGTATTATTCCGACCGTTAATGGTAAACCGGTTCGTCAG<br>ATTACCGATGATGAACTGACCTTTCTGTATAACACCTTTCAGATTTTTGCACCGAGCCAGTTTCTG<br>CCGACCTGGGTTAAAGATATTCTGAGCGTTGATTATACCGATATCATGAAGATTCTGAGCAAGAGC<br>ATTGAGAAAATGCAGAGCGATACCCAAGAAGCAAACGATATTGTTACCCTGGCAAATCTGCAGTA<br>TAACGGTAGCACACCGGCAGATGCATTTGAAACCAAAGTTACCAACATTATCGATCGCCTGAATAA<br>CAACGGCATCCACATTAATAACAAAGTTGCCTGTCAGCTGATTATGCGTGGTCTGAGCGGTGAAT<br>ACAAATTTCTGCGTTATACCCGTCATCGTCATCTGAATATGACCGTTGCAGAACTGTTTCTGGATA<br>TTCATGCCATCTATGAAGAACAGCAGGGTAGCCGTAATCCG
Ty1c CA (M-V169-N355)(M259L)(F323S)	ATGGTTCGTCCGCCTCCGATGCTGACCAGTCCGAATGATTTTCCGAATTGGGTTAAAACCTATATC<br>AAATTCCTGCAGAATAGCAACCTTGGTGGTATTATTCCGACCGTTAATGGTAAACCGGTTCGTCAG<br>ATTACCGATGATGAACTGACCTTTCTGTATAACACCTTTCAGATTTTTGCACCGAGCCAGTTTCTG<br>CCGACCTGGGTTAAAGATATTCTGAGCGTTGATTATACCGATATCATGAAGATTCTGAGCAAGAGC<br>ATTGAGAAACTGCAGAGCGATACCCAAGAAGCAAACGATATTGTTACCCTGGCAAATCTGCAGTA<br>TAACGGTAGCACACCGGCAGATGCATTTGAAACCAAAGTTACCAACATTATCGATCGCCTGAATAA<br>CAACGGCATCCACATTAATAACAAAGTTGCCTGTCAGCTGATTATGCGTGGTCTGAGCGGTGAAT<br>ACAAATCTCTGCGTTATACCCGTCATCGTCATCTGAATATGACCGTTGCAGAACTGTTTCTGGATA<br>TTCATGCCATCTATGAAGAACAGCAGGGTAGCCGTAATCCG
Ty1c CA (M-V169-N355)- VTF (M259L)(F323S)	ATGGTTCGTCCGCCTCCGATGCTGACCAGTCCGAATGATTTTCCGAATTGGGTTAAAACCTATATC<br>AAATTCCTGCAGAATAGCAACCTTGGTGGTATTATTCCGACCGTTAATGGTAAACCGGTTCGTCAG<br>ATTACCGATGATGAACTGACCTTTCTGTATAACACCTTTCAGATTTTTGCACCGAGCCAGTTTCTG<br>CCGACCTGGGTTAAAGATATTCTGAGCGTTGATTATACCGATATCATGAAGATTCTGAGCAAGAGC<br>ATTGAGAAACTGCAGAGCGATACCCAAGAAGTTAACGATATTACCACCCTGGCAAATCTGCAGTA<br>TAACGGTAGCACACCGGCAGATGCATTTGAAACCAAAGTTACCAACATTATCGATCGCCTGAATAA<br>CAACGGCATCCACATTAATAACAAAGTTGCCTGTCAGTTTATTATGCGTGGTCTGAGCGGTGAATA<br>CAAATCTCTGCGTTATACCCGTCATCGTCATCTGAATATGACCGTTGCAGAACTGTTTCTGGATAT<br>TCATGCCATCTATGAAGAACAGCAGGGTAGCCGTAATCCG

†base changes to create mutations are shown in red.
