# Supplementary material for: Probing the molecular determinants of Ty1 retrotransposon restriction specificity in yeast
Source: PLoS Genet. 2025 Oct 9;21(10):e1011898. doi: 10.1371/journal.pgen.1011898 (PMC12530519; doi:10.1371/journal.pgen.1011898)
Supplement: S7 Table — (PDF) [file pgen.1011898.s012.pdf]

S7 Table. Cloning and mutagenesis primers

Construct		Primer (5'-3')†
Ty1' CA-CTD-PET22b	FWD	CCGGAATTC**CATATG**CAGAGCGATACCCAAGAAGTG
Ty1' CA-CTD-PET22b	REV	CGCCAAGCTTTCA**CTCGAG**CGGCTGTTCTTCATACATGCTGTG
Drt2m-PET22b	FWD	CCGGAATTC**CATATG**CAGAGCGATACCCAAGAAGTG
Drt2m-PET22b	REV	CGCCAAGCTTTCA**CTCGAG**CGGACGACGCCATTCTTGCTGTTC
p18m-PET22b	FWD	CCGGAATTC**CATATG**CAGAGCGATACCCAAGAAGCA
p18m-PET22b	REV	CGCCAAGCTTTCA**CTCGAG**CGGATTACGGCTACCCTGCTGTTC
TY1c CA(M-V169-N355)-PET22b	FWD	CCGGAATTC**CATATG**GTTCGTCCGCCTCCGATGCTG
TY1c CA(M-V169-N355)-PET22b	REV	CGCCAAGCTTTCA**CTCGAG**CGGATTACGGCTACCCTGCTGTTC
Ty1' CA-CTD (F323S)	FWD	AGCGGTGAATACAAATCTCTGCGTTATGCACGT
Ty1' CA-CTD (F323S)	REV	ACGTGCATAACGCAGAGATTTGTATTCACCGCT
Drt2m (F323S)	FWD	AGCGGTGAATATCGTTCTCTGCGTTATGCACGT
Drt2m (F323S)	REV	ACGTGCATAACGCAGAGAACGATATTCACCGCT
Drt2m (Y326S)	FWD	TATCGTTTTCTGCGTTCTGCACGTTATCGCTGC
Drt2m (Y326S	REV	GCAGCGATAACGTGCAGAACGCAGAAAACGATA
Drt2m Y329S)	FWD	CTGCGTTATGCACGTTCTCGCTGCATTAATATG
Drt2m (Y329S)	REV	CATATTAATGCAGCGAGAACGTGCATAACGCAG
p18m (F323S)	FWD	AGCGGTGAATACAAATCTCTGCGTTATACCCGT
p18m (F323S)	REV	ACGGGTATAACGCAGAGATTTGTATTCACCGCT
TY1c CA(M-V169-N355) (M259L)	FWD	AAGAGCATTGAGAAACTGCAGAGCGATACCCAA
TY1c CA(M-V169-N355) (M259L)	REV	TTGGGTATCGCTCTGCAGTTTCTCAATGCTCTT
TY1c CA(M-V169-N355) (F323S)	FWD	AGCGGTGAATACAAATCTCTGCGTTATACCCGT
TY1c CA(M-V169-N355) (F323S)	REV	ACGGGTATAACGCAGAGATTTGTATTCACCGCT

†Restriction sites used for cloning are underlined; †base changes to create mutations are shown in red.
