# Supplementary material for: Probing the molecular determinants of Ty1 retrotransposon restriction specificity in yeast
Source: PLoS Genet. 2025 Oct 9;21(10):e1011898. doi: 10.1371/journal.pgen.1011898 (PMC12530519; doi:10.1371/journal.pgen.1011898)

Fig. S2A raw western blot (chemiluminescence)

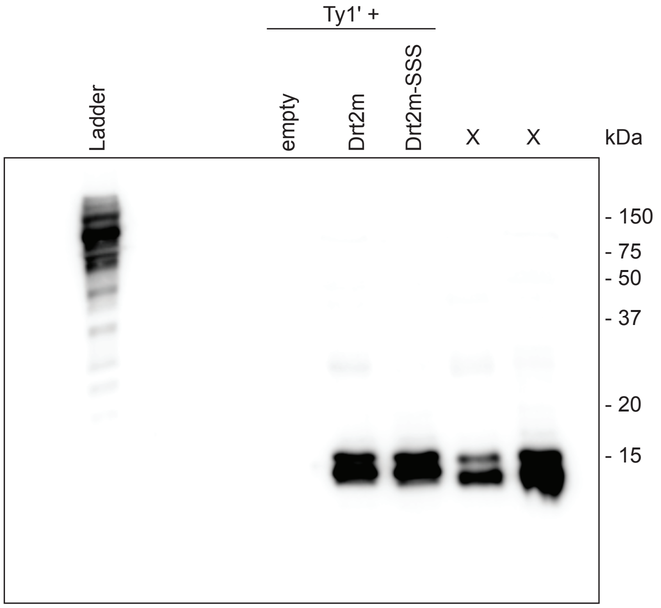


Restriction Factor (RF); α-His

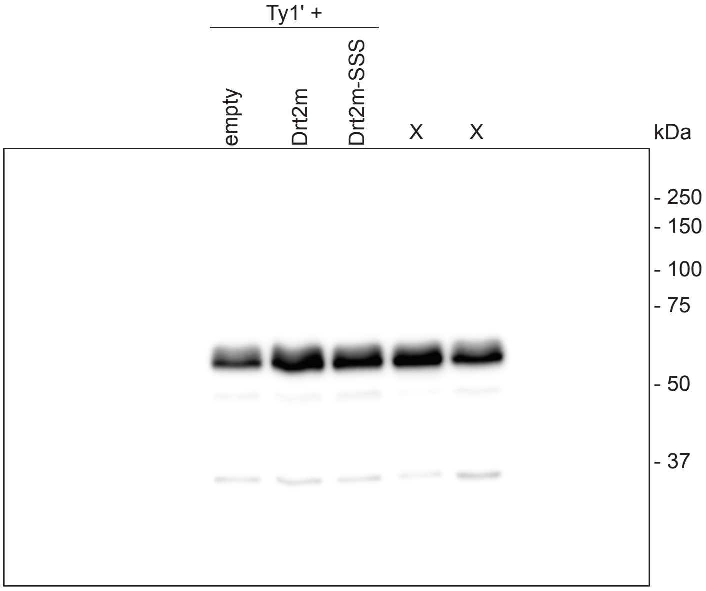


Gag; α-p18'

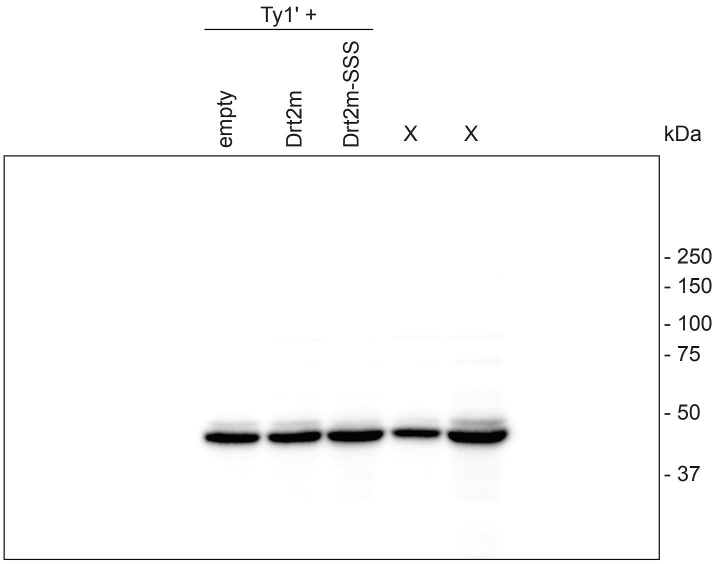


Pgk1; α-Pgk1

Fig. 5B Ty1c strains raw western blot (chemiluminescence)

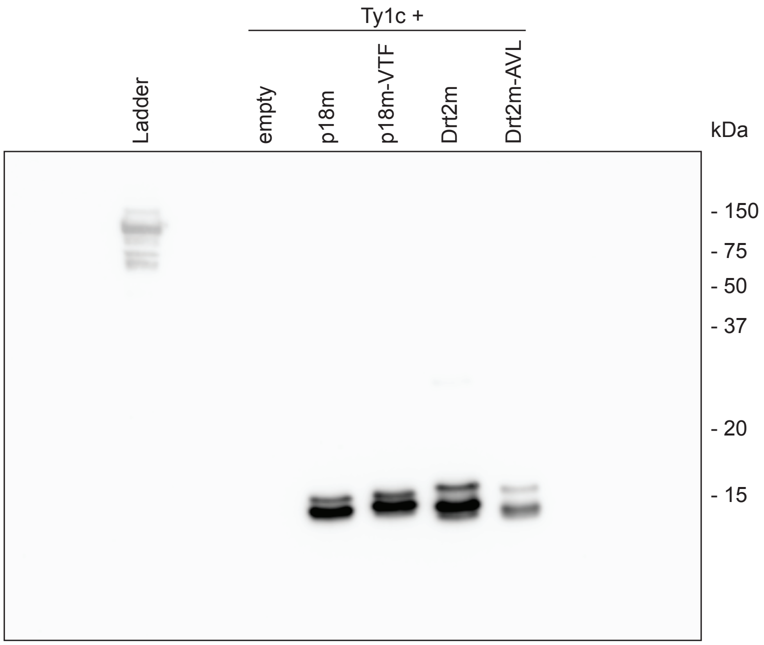


Restriction Factor (RF); α-His

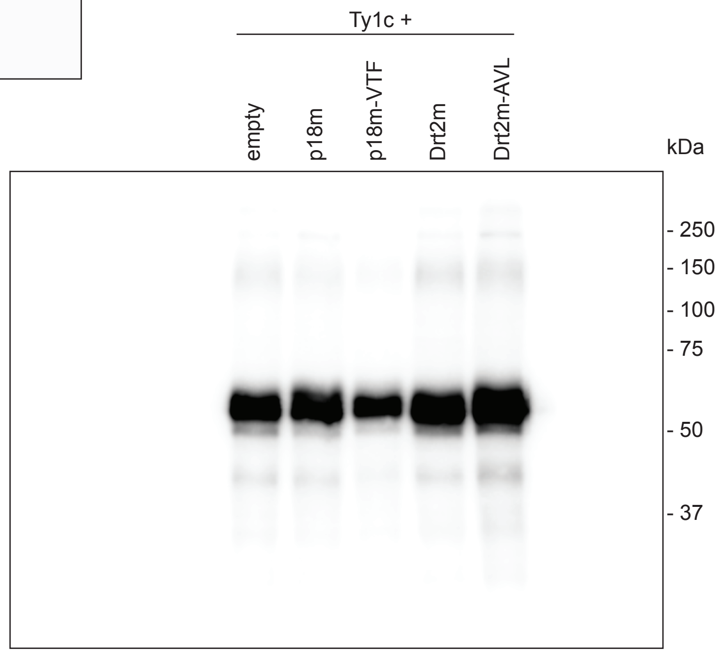


Gag; α-TY

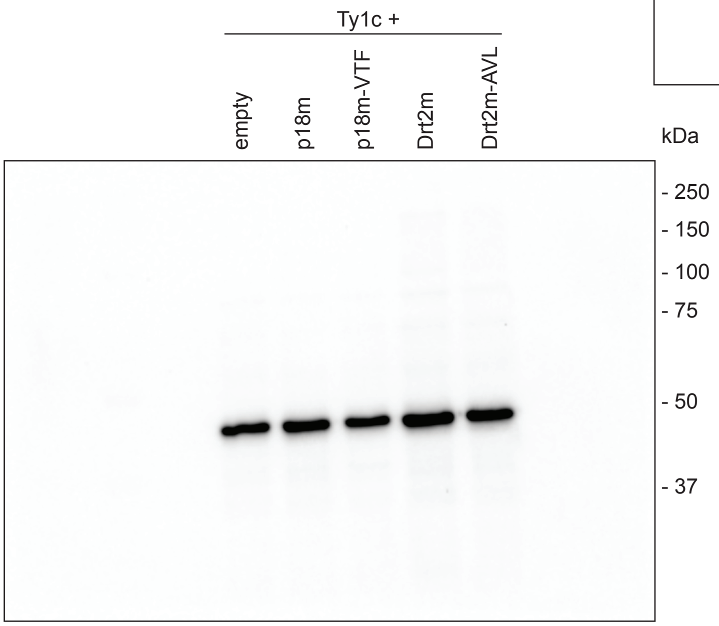


Pgk1; α-Pgk1

Fig. 5B Ty1c-VTF strains raw western blot (chemiluminescence)

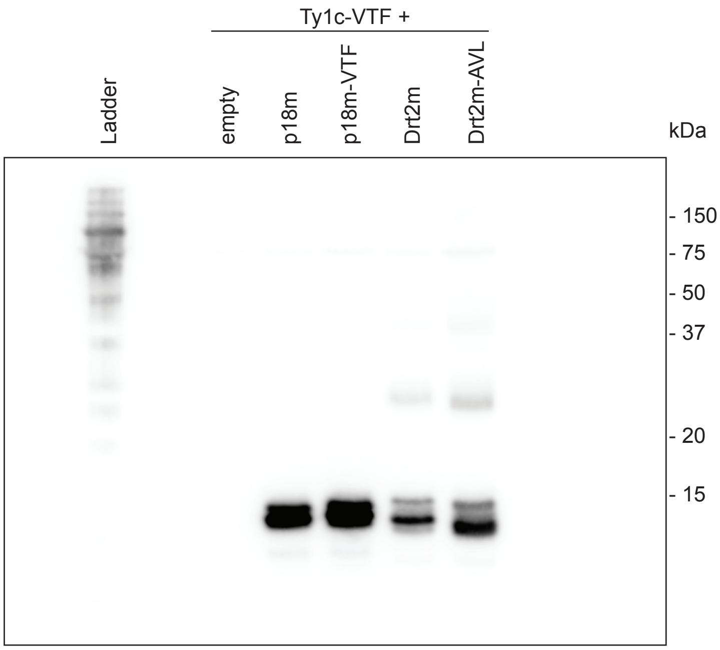


Restriction Factor (RF); α-His

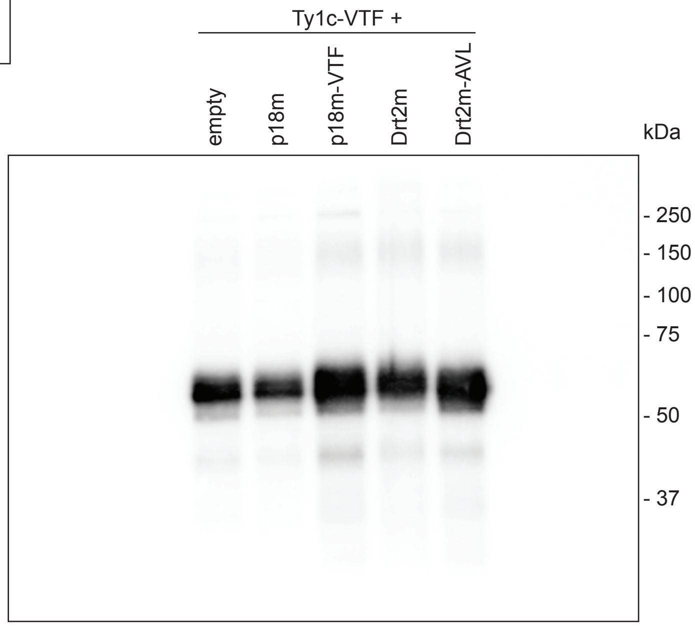


Gag; α-TY

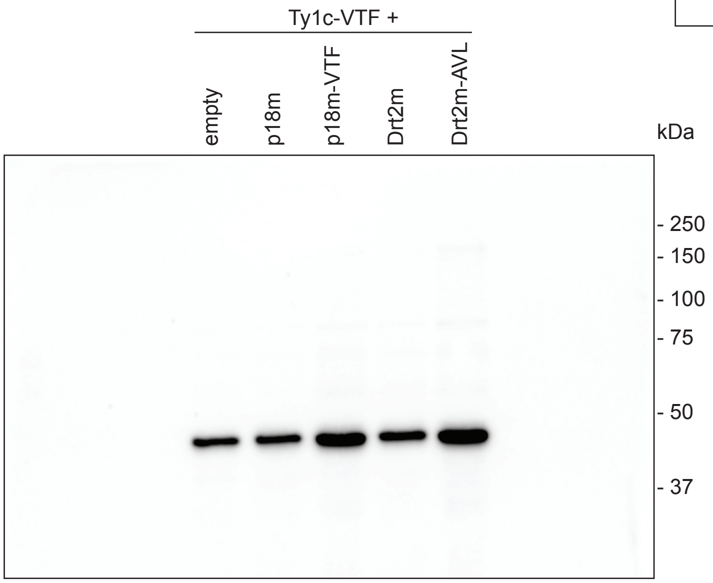


Pgk1; α-Pgk1

Fig. 5B Ty1' strains raw western blot (chemiluminescence)

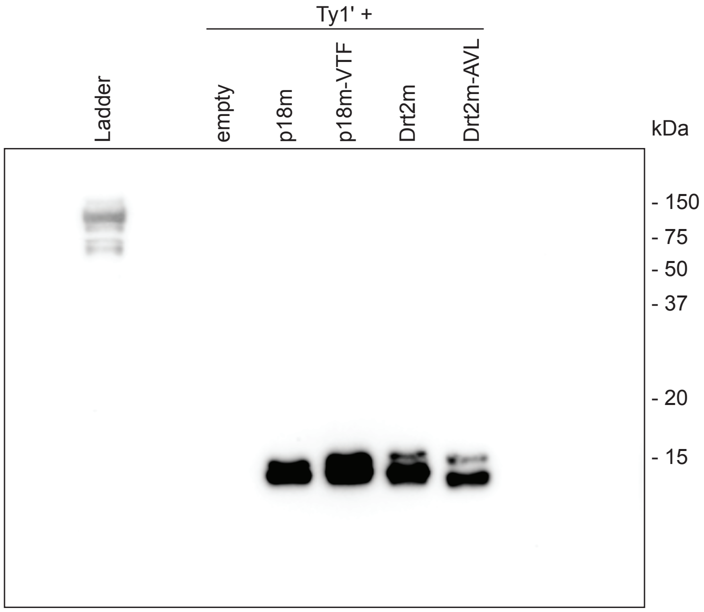


Restriction Factor (RF); α-His

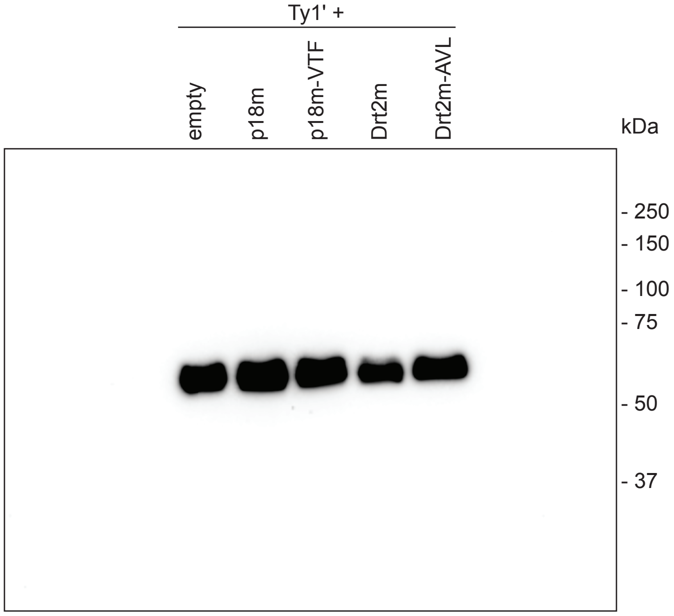


Gag; α-p18'

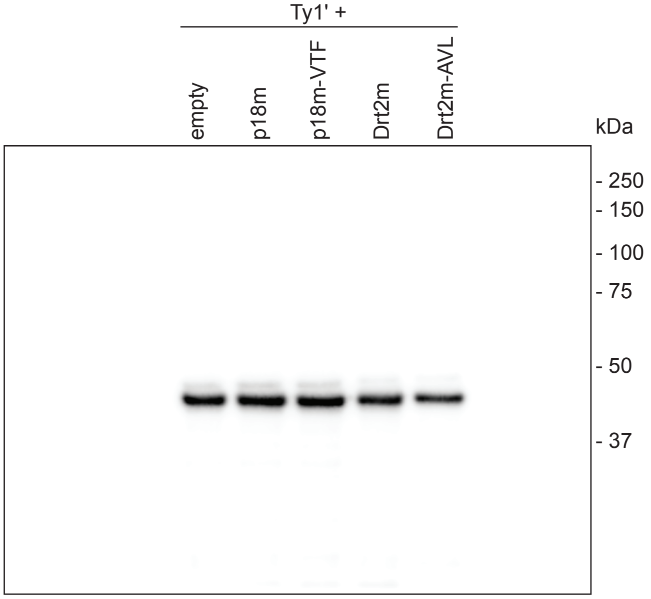


Pgk1; α-Pgk1

Fig. 5B Ty1'-AVL strains raw western blot (chemiluminescence)

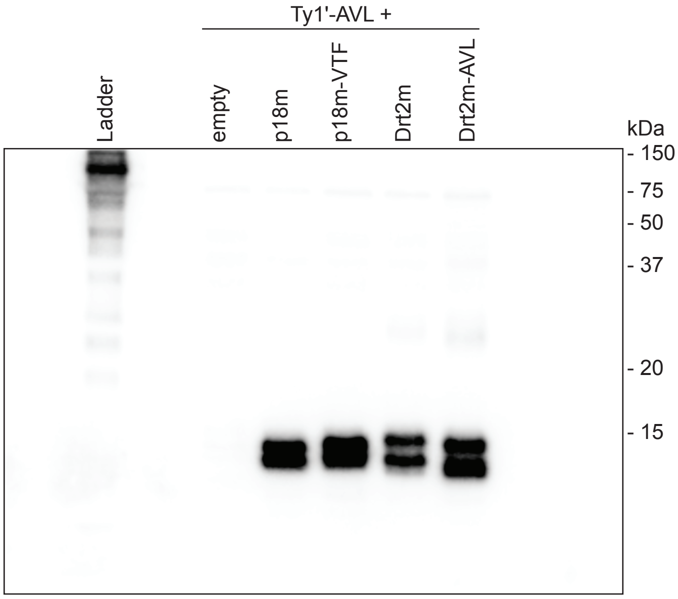


Restriction Factor (RF); α-His

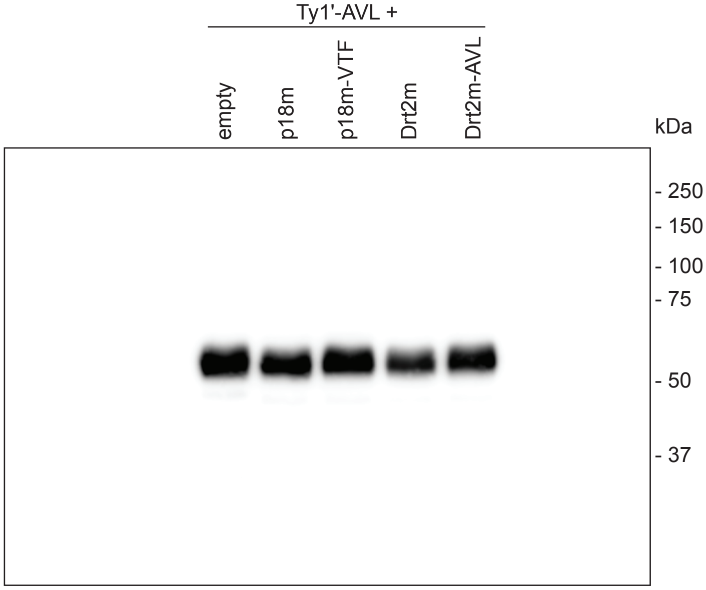


Gag; α-p18'

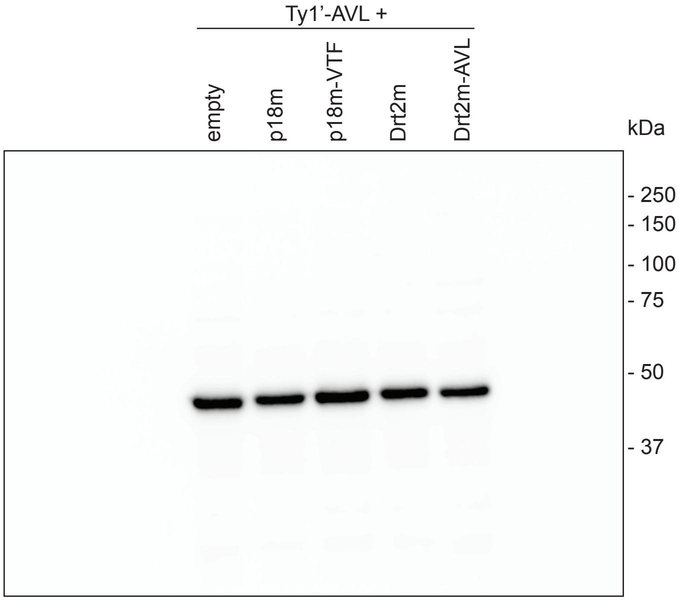


Pgk1; α-Pgk1

Fig. 6 raw western blot (chemiluminescence)

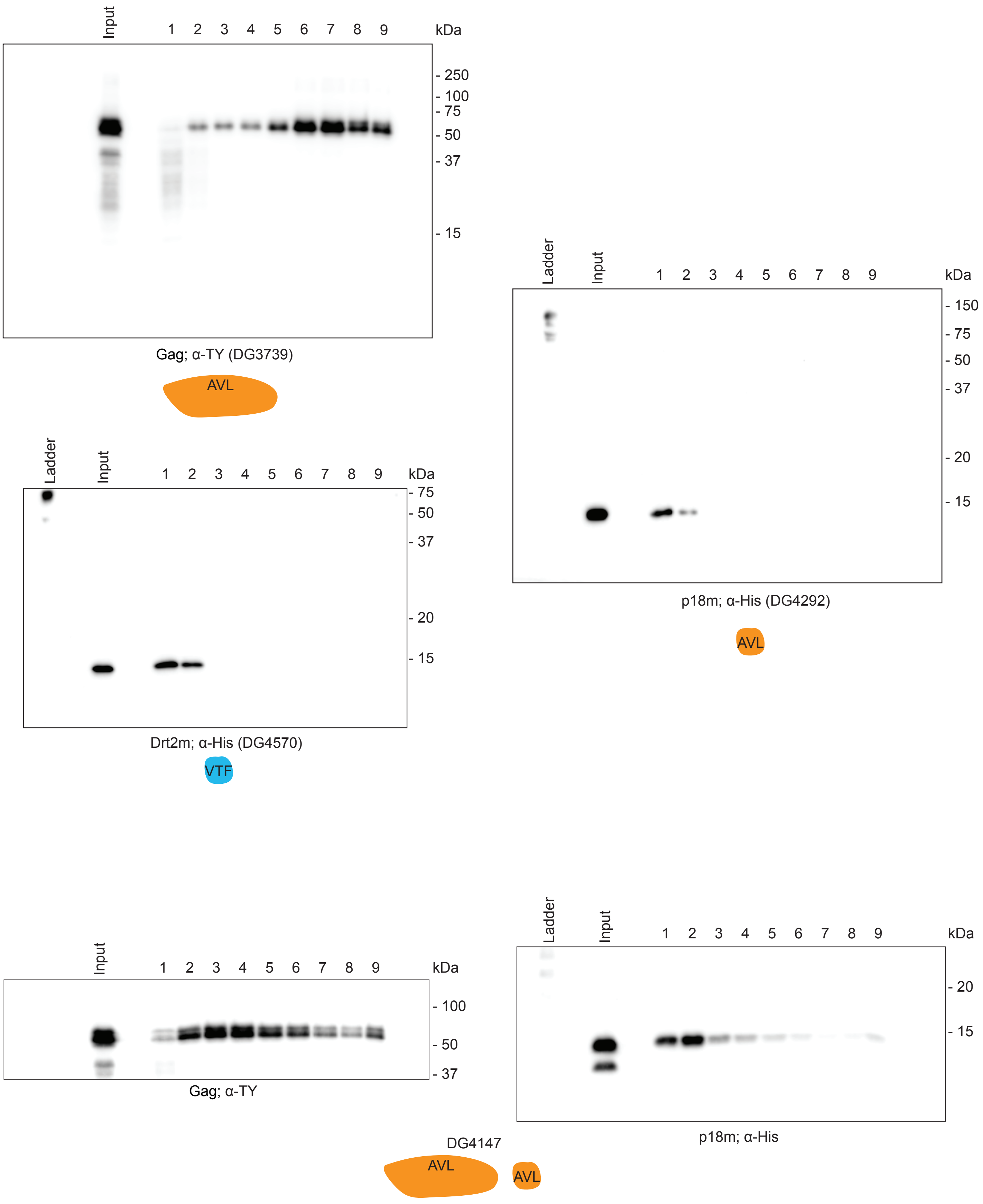

Fig. 6 raw western blot (chemiluminescence)

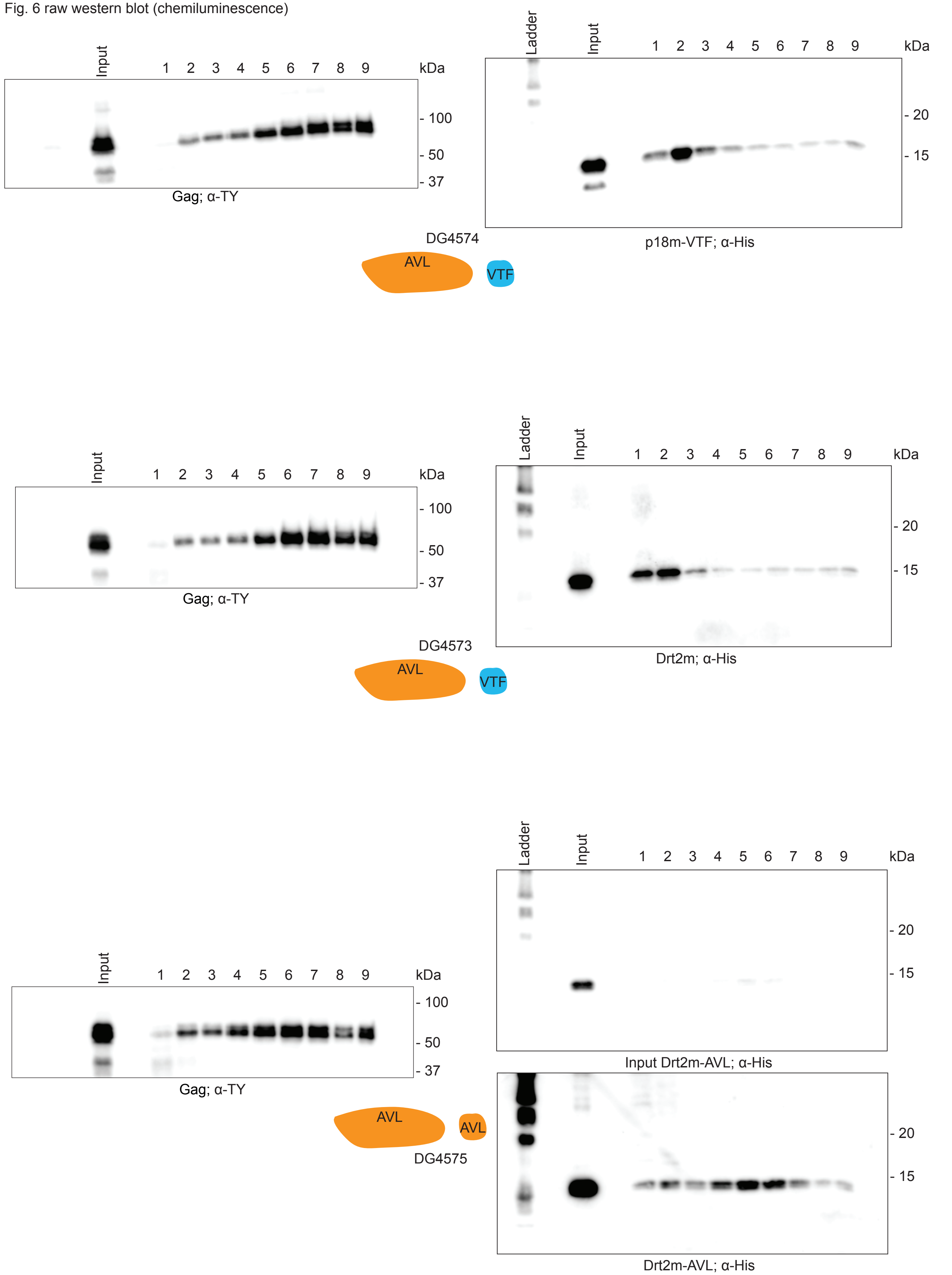

Supplement: S1 Raw Images — (PDF) [file pgen.1011898.s013.pdf]
