# Supplementary material for: Probing the molecular determinants of Ty1 retrotransposon restriction specificity in yeast
Source: PLoS Genet. 2025 Oct 9;21(10):e1011898. doi: 10.1371/journal.pgen.1011898 (PMC12530519; doi:10.1371/journal.pgen.1011898)
Supplement: Attachment — Submitted filename: Beckwith_et_al_Response to Reviewers_250925.docx [file pgen.1011898.s015.docx]

**Response to Reviewers: manuscript PGENETICS-D-25-00848, Beckwith et al 2025**

***Reviewer-1 (Remarks to Author):***

*The study is well-written, employs detailed and rigorous methodology, and combined multiple biophysical and genetic approaches to corroborate the proposed mechanism. Very elegant and convincing work that will be of broad interest to readers studying retrotransposon/retroviral control as well as those studying protein structure-function relationships.*

We thank Reviewer-1 for their comments and appreciation of the manuscript.

*Comments*

*The introduction provides a useful and detailed primer on the mechanisms of Ty1c and Ty1’ restriction mechanisms necessary for understanding the proposed study. As a reader, I was also seeking a brief introduction of literature with analogous restriction systems beyond the Ty elements in S. cerevisiae to better understand the context for the study and potential for broader implications. In the discussion, authors highlight the Fv1 restriction system for murine leukemia virus which is described as analogous to the Ty1/p22 and Ty1’Drt2 system, where AA substitutions clustered in the capsid can also alter restriction specificity. I think a nod to this body of literature in the introduction, instead of the discussion alone, is appropriate and further provides a basis for the rationale of the approach.*

We have now included additional sentences in the introduction, lines 111-113, to introduce the broader context of restriction through capsid recognition across other systems.

*Similarly, I was curious about other Ty elements (Ty2, Ty4, Ty5) and Ty3 families. Lines 379-318 in the discussion state “whether there is cross-restriction between Ty2 and certain Ty1 subfamilies or between other Ty elements remains to be determined.” Has the mechanism of restriction for these elements been determined or relevant proteins/domains defined that can be compared to Ty1 restriction? A more detailed explanation would be helpful.*

We agree that this is a very interesting question. Mechanisms of restriction have not been investigated in the other Ty elements, but they similarly contain capsid domains that could be subject to lattice-poisoning restriction. We hope to address these questions in future studies.

*Does the S. cerevisiae background strain used for in vivo experiments contain Ty1c or Ty1’ genomic copies? If so, would this influence the Ty1c and Ty1’ reporter-based mobility/restriction (for example p22/p18 production from genomic copies)?*

The *S. cerevisiae* (PMID: **32084126)** and *S. paradoxus* (PMID: **35049349**) strains used for in vivo experiments have been sequenced and do not contain any full-length Ty1c or Ty1' elements. These strains have been previously used to study p22/p18 and Drt2 and were specifically chosen to avoid endogenous p22/p18 expression which may confound the genetic studies. The description of the strains in the first paragraph of Results has been updated to make this Ty-less background explicit.

*For the plasmids with reporter constructs and restriction factors introduced, is the approximate copy number per cell known and whether plasmids are stably maintained? A brief mention of this would be helpful, though the consistency in the fold-restriction data across experiments suggests stable maintenance.*

Cells were grown in media selecting for plasmid maintenance by complementing auxotrophic chromosomal mutations by markers on each plasmid. In other studies (e.g., PMID: 37459521), we have observed a low frequency of plasmid loss when cells are not grown on selective media, although we have not quantified this frequency. While plasmid copy number has not been measured in the Ty-less strains used in this study, they have been measured in the common lab strain BY4741 (PMID: 23107142). Centromere plasmids are maintained at 2-5 copies per cell and 2-micron plasmids are multi-copy and range from 14-34 copies per cell. The text in the Results, lines 326-328 describing these plasmids has now been updated to reflect their relative copy number.

*Minor suggested edits:*

*The following terms may not be familiar to most readers – suggest briefly defining when first introduced:*

*Line 30, 82: ‘endogenized’ restriction factor*

*Line 72: ‘sub-genomic’*

To aid clarity, the text referring to “endogenized gene” has been replaced with “a gene present at a fixed chromosomal locus” and “sub-genomic replaced with “initiated within *GAG*”.

*Line 120: include Gag in this sentence for clarity: “and the p18m region of Ty1' Gag is 92.8%...”*

This sentence, now line 157, has been revised for clarity

*Fig 1 E and F – is there a reason for the dotted lines outlining the images? Suggest no outline or solid outline instead.*

The dotted outlines have been removed from the figure.

*Fig 5C: Error bars appear to be missing on final Drt2m-AVL lane.*

No retromobility events were observed in this lane, so the theoretical maximum fold restriction based on the limit of detection of the assay is plotted without error bars. This was previously only described in S1 Table but that information has now been added to the legend for Figure 5.

***Reviewer #2 (Remarks to the Author):***

*The quality of the data is high, and the manuscript is well written. The genetic and structural results provide a detailed understanding of the Ty1 restriction mechanisms. This level of detail is missing in most if not all examples of retrovirus restriction factors such as Fv1 that are domesticated fragments of Gag. The details of Ty1 restriction are thus a valuable model for understanding how assembly of that are domesticated fragments of Gag. The details of Ty1 restriction are thus a valuable model for understanding how assembly of Gag-like particles is altered. The importance of understanding this process is highlighted recently in Science (PMID: 40773553).*

We thank Dr. Levin for his review and appreciation of the manuscript.

*Detailed comments:*

*1. Line 58, I would list proteins in order of their position in Pol, PR, IN, and RT.*

Order of proteins has been switched to match the position.

*2. Line 62, Is it clear that reverse transcription of Ty1 occurs in the cytoplasm. This view has changed with HIV-1 with strong evidence reverse transcription occurs in the nucleus.*

We have added the following to this section of the Introduction, with additional references: Unlike HIV-1 reverse transcription which is completed within the nucleus, available biochemical and genetic analyses suggest that Ty1 reverse transcription occurs in cytoplasmic VLPs.

*3. Line 99, what is the relationship between the presence of DRT2 and Ty1' across populations of cerevisiae? Do strains lacking Ty1' have DRT2*?

*DRT2* is widespread and is present in strains with and without Ty1' elements. There have been multiple gene loss events leading to strains without *DRT2*, and these strains largely contain Ty1' (including S288C). The prevalence of *DRT2* in diverse strains and its absence in the S288C reference strain is now stated in the first paragraph of the results section.

*4. Line 118, Fig. S1 title indicates that Ty1c is also shown. But it’s not. I understand that p18m is the same sequence as Ty1c so why not label it with both names. p18m and Ty1c.*

Fig S1 has been updated to include both p18m and Ty1c

*5. Line 137 and throughout. It would be best to call this CA-CTD. You do list the segment M259-Q351, but it would be best to indicate it’s actually the C-terminal half of CA.*

We have replaced CA(M259-Q351) with CA-CTD throughout the manuscript and supporting information, leaving the Gag coordinates at the first mention in the Results and in the Methods.

*6. Line 172, Fig 2D and E appear to be of the monomer not the dimer. I would like to see these residues in a dimer making clear it’s the interface in an image like Fig. 2A. In such a drawing you can highlight the dimer interactions of the 3 key residues. It would be easier to understand and would complement the blowup shown in Figs 2F and G.*

In our first publication (PMID: 34552077) we did use the full dimer representation to try to display the contacts across the interface. However, we became aware that the image becomes very crowded and nearly impossible to see all of the residues at the front (α1) and rear (α3) of the interface. Therefore, having tried both ways we feel the representation focussed on looking into the interface of one monomer is better able to show all of the residues involved. We can see some need for further clarity and so have now improved the legend to state the viewer is looking perpendicular and into a single monomer of the Dimer in panels **D** and **E** and with the same view of the dimer as **A** & **B** in panels F and G.

*7. Line 175, It would help to ref. Fig. 2 F and G here. Helix alpha 3 is not labelled in Fig 2F or 2G.*

The references to Fig 2F and 2G have been moved in the text and the helices (α1 and α3) are now labelled in 2F & 2G.

*8. Line 217, Please present a general concept of SEC-MALLS.*

We have now added an additional explanatory sentence regarding the SEC-MALLS method to the first paragraph of the SEC-MALLS section of the results.

*9. Line 246, Reference Table 1 here.*

Table 1 is now also referenced on Line 305

*10. Line 250, These exchange factors show a clear trend at the higher levels. But the exchanges are not obvious in Fig. 4 for the lower levels such as the difference between 6.2% of Drt2m-AVL with CA. vs the 2.3% of Drt2m-AVL with CA-VTF. The former represents exchange while the latter no exchange. Are there statistics that can be applied that can distinguish the exchangers from the nonexchangers?*

This is difficult to really metric, but we have made some attempt. We can first derive a baseline for the sum fraction residual of runs of the un-mixed samples or non-exchanging pairs. This contains noise from imperfections in the chromatographic peaks, baseline drift and from the chromatogram subtraction itself. Even using precision HPLC to carry out the runs the average background sum fraction residual we obtain from these measurements, and the processing method is 2.2 % for non-exchanging pairs. To do the analysis we then looked at the sum fraction residual for all pairs and compared to this background. Those that give a sum fraction residual greater than 2x background (4.4 %) are scored as exchanging with the underline in Table 1. This methodology is stated in the Table 1 (upper) footnote, but we have now also added explanatory text to the method section. In Table 1 (lower) the degree of exchange is derived from the same data but just using ranking score of 3 down to 0 using chosen criteria of > 5x, >2x, >1.3x and <1.3x background. We are confident with the data. However, for clarity we have now amended the results section to point out that for the Ty1c CA-VTF(F323S) - Drt2m(SSS) pair this value assigned to weak exchange is close to baseline.

*11. Line 280, Although most of the differences in Fig 5 are obviously significant, there is one pair that could use a statistical test. Ty1'-AVL comparing p18m to Drt2M. Does p18m show a statistically higher fold restriction than the Drt2m?*

Yes, against Ty1'-AVL, p18m fold restriction is over twice as strong as Drt2m (p = 0.00015). The term “significantly” has now been added to the text.

*12. Line 309, If DRT2 is fixed, is Ty1' older than Ty1? What is the context of the Drt2 gene. Is there a remnant of the LTR, does it have other recognizable parts of Ty1. Does the promoter of DRT2 share regulatory sequence with Ty1' that is independent from Ty1 expression? An interesting evolutionary question is why did p18 evolve to be self-limiting while DRT2 is domesticated?*

Ty1' is the ancestral element and Ty1 diverged when a new Gag sequence was acquired from a horizontal transfer event from *S. paradoxus* (original line 359). While it is unclear what regulatory sequences may function at *DRT2*, gene expression appears to be driven from a Ty1i-like transcription start site and perhaps a minor transcriptional product from an upstream Ty2 solo LTR transcription start site. We agree that the difference between p18 and Drt2 is an interesting evolutionary question, and we speculate it may be related to the greater intrinsic transposition activity of Ty1c. Further description of the context of the *DRT2* locus has been added to the Discussion, paragraph 5.

*13. Line 397, should reference PMID: 40773553.*

We thank the reviewer for this suggestion; the review was published after submitting this manuscript, but we are pleased to include it now in the discussion as (Ref 40). In addition, we have also updated Reference 42 to a more pertinent study (PMID: 24603659).

***Reviewer #3 (Remarks to the Author):***

*Overall, I found this article to be solid and interesting. The experiments are clear and the data corroborate the conclusions drawn. Perhaps the results are not entirely unexpected, but the fact that three amino acids are necessary and sufficient to ensure restriction specificity while maintaining a similar 3D structure between the two restriction factors is nonetheless remarkable. As the authors point out in their discussion and argue with examples in the literature, this supports the idea that such a restriction mechanism may have*

*emerged repeatedly to “self-regulate” other retroelements.*

We thank Reviewer-3 for their insightful assessment of the manuscript.

*Comments.*

*Figure 1 and the text in the results section that refers to it.*

*The text lacks details to understand the retromobility assay. First, retromobility is a term specific to Ty retrotransposons and should be explained. A brief explanation of the his3-AI system would also help those unfamiliar with it. Second, it should be clarified in the text (at least in the legend of Figure 1) that the assay is performed in a Ty1c-less context to avoid Ty1c restriction by endogenous Ty1c elements.*

Additional explanations have been added to the first paragraph of the Results section to clarify these points.

*The restriction of Ty1’ by DRT2 is quite weak (Fig 1C). This could mean that Drt2 is limiting in fully restricting Ty1’. What happens when DRT2 is overexpressed?*

Endogenous Drt2 expression is very low, partially explaining weak restriction (Hannon-Hatfield et al., MBE 2024). Overexpressing Drt2 results in 30-fold restriction in a *S. cerevisiae* background (Hannon-Hatfield et al., MBE 2024) or over 100-fold restriction in an *S. paradoxus* background (Fig 5). This is still much weaker restriction than p18m achieves using the same overexpression promoter and plasmid and we speculate on a role for differences of aromatic residues in the dimer-2 interface in the Discussion, paragraph-4.

*Given that the retromobility of Ty1c is several orders of magnitude higher than that of Ty1’, we cannot exclude from these data that Drt2 levels are limiting in restricting Ty1c. Even if the subsequent data indicate that this is mainly due to sequence specificity, this hypothesis should at least be mentioned at this stage of the manuscript.*

We agree that at this stage of the study, Drt2 levels could be limiting restriction, and have now added this qualifier in the first paragraph of the Results text.

*It could also be relevant to show data comparing the RNA and Gag levels of Ty1c and Ty1’.*

In this work, GAL1-promoted Ty1 and restriction factor expression in a Ty-less background was used to analyze specific interactions between a complete element and the restriction factor. Moving forward, we are interested in discovering new interactions across Ty1-Ty5 element families where we believe comparing RNA levels in these future studies will be important. With respect to Gag protein levels, presently, comparing the level of Ty1c and Ty1’ Gag is complicated by antibody avidity and specificity so is not really something we can undertake. Specifically, the Ty1c Gag antiserum is much stronger than the Ty1’ antiserum as reported previously for Ty1’ Gag (PMID: **38442736**) that confounds comparative measurements. In addition, given Gag must assemble through multiple protein-protein interfaces we are concerned that the inclusion of epitope tags for detection risks also altering Gag function or structure.

*Fig 1C and Table S1: x107 should be x10-7*

This negative sign has been added to the exponent in the figure and table.

*Fig 1D and the corresponding section “Subfamily specific residues in the Dimer-1 interface”, you should refer to the materials and methods section, which describes in more detail which sequences were used, otherwise the paragraph seems a bit vague.*

A reference to the Methods section has now been added

*Figure S1, and Tables S4 and S5: It is not easy to understand which strains have been used in the retromobility assays. Could you add a column in Table S4 to indicate in which experiments the strains have been used?*

A column has been added to S4 Table indicating the figure(s) each strain is used in.

*Figure 2 and the text in the results section that refers to it.*

*Line 120: p18m should be Drt2-like? In the section “Subfamily specific residues in the Dimer-1 interface”, you should refer to the materials and methods section, which describes in more detail which sequences were used, otherwise it's difficult to follow.*

The text has been updated to “p18m-equivalent region of Ty1' Gag” for clarification and a reference to the Methods section has been added.

*Fig S2B and S2C: Ty1 should be Ty1c in the graphs and the drawings.*

This has been corrected

*Fig S2C: x107 should be x10-7*

This has been corrected

*Figure 4 and Table 1. Drt2m(SSS) seems to make weak exchanges not only with Ty1-CA(F323S) VTF but also Ty1-CA(F323S). Could you discuss this point?*

In making these measurements we derived a background sum fraction residual of 2.2 % from non-exchanging restriction factor CA pairs that we applied as baseline in the analysis of all pairs. The degree of exchange we observe for exchanging pairs varies from 25.4 % for Ty1c CA(F323S) - p18m(F323S) (11 x background), through 6.2 % for Ty1c CA(F323S) - Drt2m-AVL(SSS) (3x background) to only 3.2% for Ty1c CA-VTF(F323S) - Drt2m(SSS) (1.5x background). Given these values, our interpretation of the Drt2m(SSS) data is rather than there being weak exchange with both Ty1c CA-VTF(F323S) and Ty1c CA(F323S), there is no exchange (background value) with Ty1c CA(F323S) and that with Ty1c CA-VTF(F323S) the value is above the background but not enough to really call. To engage with this point in the text of the results section, we have now added a qualifier that the Ty1c CA-VTF(F323S) - Drt2m(SSS) is close to the baseline value. In addition, we have amended the Table footnotes, so it is clear what criteria we have used and added an explanatory sentence to the methods. Nonetheless, the *in vitro* exchange measurements do match well with the restriction data.

We agree the non-reciprocity in the system is intriguing and it is tempting to speculate that there might be secondary effects on the homo- heterodimer on and off rates from residue differences outside of the Dimer-1 interface that effect the *in vitro* exchange. However, presently we have no evidence to support these ideas and although it would be interesting to investigate these phenomena in a protein structure and dynamics study, we believe including these speculations in the present discussion would risk detracting from the main message of the paper. (Please also see the response to Reviewer-2, point 10).

*Figure 5 and Figure 6.*

*These are very nice data. Fig 5B. The Western blots seem to indicate that Drt2m-AVL is more abundant/stable in the presence of Ty1'-AVL. Is this observation reproducible? Do you have a hypothesis based on the other results?*

Yes, Drt2m-AVL was repeatably observed as somewhat less abundant/less stable even when expressed in the absence of any transposon but had higher levels when co-expressed with Ty1'-AVL. Our hypothesis, although speculative, is that it that Drt2m-AVL becomes stabilized when binding the homotypic dimer-1 interface of Ty1'-AVL and may even be further stabilized by dimer-2 interactions that are missing in Ty1c.

*Minor comments:*

*Line 49: “cerevisiae” must be in italic*

This has been corrected, now line 67.

*Line 51: Why ref. 7?*

We agree there is a better source for transposable element content of yeast genomes and replaced this reference with PMID: 9582191.

*Line 100: “… in diverse strains”, Please, add “but absent in classical laboratory strains”.*

The text has been amended to “but absent in the S288C reference strain”. *DRT2* is present in laboratory strains SK1, Y55 and other common laboratory strains such as W303 have not been evaluated.

*Line 110-114. The sentence needs to be clarified.*

This sentence, now line 147-150, has been revised for clarity.

*Line 120: p18m should be p18m-like or Drt2-like?*

The text has been updated to “p18m-equivalent region of Ty1' Gag”.

*Line 146: Please, refer to Fig S2B.*

We have added a reference to Fig S2B.

*Line 178: “of the greater steric bulk”, remove one “of the”.*

The duplication has been corrected.

*Line 224: Please, refer to Fig 1E and 1F.*

We have added a reference to Fig 1E-F.
